# No Additional Benefits of Block- Over Evenly-Distributed High-Intensity Interval Training within a Polarized Microcycle

**DOI:** 10.3389/fphys.2017.00413

**Published:** 2017-06-13

**Authors:** Kerry McGawley, Elisabeth Juudas, Zuzanna Kazior, Kristoffer Ström, Eva Blomstrand, Ola Hansson, Hans-Christer Holmberg

**Affiliations:** ^1^Department of Health Sciences, Swedish Winter Sports Research Centre, Mid Sweden UniversityÖstersund, Sweden; ^2^Åstrand Laboratory, Swedish School of Sport and Health SciencesStockholm, Sweden; ^3^Diabetes and Endocrinology, Department of Clinical Sciences, Lund University Diabetes Centre, Lund UniversityMalmö, Sweden

**Keywords:** cross-country skiing, endurance, junior athletes, muscle, periodization, recovery, stress, training load

## Abstract

**Introduction:** The current study aimed to investigate the responses to block- versus evenly-distributed high-intensity interval training (HIT) within a polarized microcycle.

**Methods:** Twenty well-trained junior cross-country skiers (10 males, age 17.6 ± 1.5 and 10 females, age 17.3 ± 1.5) completed two, 3-week periods of training (EVEN and BLOCK) in a randomized, crossover-design study. In EVEN, 3 HIT sessions (5 × 4-min of diagonal-stride roller-skiing) were completed at a maximal sustainable intensity each week while low-intensity training (LIT) was distributed evenly around the HIT. In BLOCK, the same 9 HIT sessions were completed in the second week while only LIT was completed in the first and third weeks. Heart rate (HR), session ratings of perceived exertion (sRPE), and perceived recovery (pREC) were recorded for all HIT and LIT sessions, while distance covered was recorded for each HIT interval. The recovery-stress questionnaire for athletes (RESTQ-Sport) was completed weekly. Before and after EVEN and BLOCK, resting saliva and muscle samples were collected and an incremental test and 600-m time-trial (TT) were completed.

**Results:** Pre- to post-testing revealed no significant differences between EVEN and BLOCK for changes in resting salivary cortisol, testosterone, or IgA, or for changes in muscle capillary density, fiber area, fiber composition, enzyme activity (CS, HAD, and PFK) or the protein content of VEGF or PGC-1α. Neither were any differences observed in the changes in skiing economy, $\dot{\text{V}}{\text{O}}_{2}\text{max}$ or 600-m time-trial performance between interventions. These findings were coupled with no significant differences between EVEN and BLOCK for distance covered during HIT, summated HR zone scores, total sRPE training load, overall pREC or overall recovery-stress state. However, 600-m TT performance improved from pre- to post-training, irrespective of intervention (*P* = 0.003), and a number of hormonal and muscle biopsy markers were also significantly altered post-training (*P* < 0.05).

**Discussion:** The current study shows that well-trained junior cross-country skiers are able to complete 9 HIT sessions within 1 week without compromising total work done and without experiencing greater stress or reduced recovery over a 3-week polarized microcycle. However, the findings do not support block-distributed HIT as a superior method to a more even distribution of HIT in terms of enhancing physiological or performance adaptions.

## Introduction

Various theories pertaining to training periodization (i.e., the division of an athlete's seasonal training into smaller units and periods of time) have been discussed in the scientific literature (see Issurin, 2010 for a comprehensive review). However, these theories have been criticized for lacking an evidence base (Kiely, 2012), which seems to be due, at least in part, to the logistical challenges associated with comparing different forms of training within athlete groups. In one study that reported the effects of two different training periodization models used over two seasons with elite kayakers (García-Pallarés et al., 2010), rigorous scientific control was limited due to the applied and longitudinal nature of the study. The outcomes of group studies are further complicated by the variability of individual responses to long-term training programs (Mann et al., 2014). On the other hand, case studies of effective organizational strategies over seasonal periods (e.g., Støren et al., 2012) provide limited support for general use.

Experimental studies designed to investigate the effects of different training organization models on physiological and performance adaptations are typically limited to short intervention periods. This results in a need for potent training stimuli, particularly where athlete populations are concerned. One such method used to develop aerobic power and performance has been to concentrate a large number of high-intensity interval training (HIT) sessions (e.g., 5–15 sessions) into a short period of time (e.g., 6–14 days) (Stølen et al., 2005; Breil et al., 2010; Wahl et al., 2013, 2014; Rønnestad et al., 2014b, 2016). This strategy has been referred to as “block training” and is based on the overload principle, with a super-compensation in selected fitness components thought to occur after a period of focused loading followed by a short recovery period (Issurin, 2010).

While HIT is considered necessary to elicit physiological and performance gains among endurance-trained athletes (Laursen et al., 2002; Iaia et al., 2009; Buchheit and Laursen, 2013a,b; Gunnarsson et al., 2013), low-intensity training (LIT) remains a fundamental component of endurance programs (Sandbakk and Holmberg, 2014). Combining these two forms of contrasting training stimuli, while performing relatively little moderate-intensity training (MIT), is referred to as polarized training (Seiler and Kjerland, 2006; Laursen, 2010). Some studies have suggested that more polarized training distributions are beneficial to endurance performance (Esteve-Lanao et al., 2007; Neal et al., 2012; Stöggl and Sperlich, 2014). Moreover, two recent studies have demonstrated improvements in $\dot{\text{V}}{\text{O}}_{2}\text{max}$, maximal power output (MPO) and power output at blood lactate concentrations of 2–4 mmol·L^−1^ among cyclists and cross-country skiers following a period of block-distributed polarized training (i.e., a series of concentrated HIT sessions followed by LIT), but not after a more traditional (even) distribution of LIT and HIT (Rønnestad et al., 2014b, 2016).

Despite these positive findings associated with blocking HIT, prescribing short periods of intensified training in this manner has also been shown to result in the development of overreaching (OR) symptoms. For example, Halson et al. (2002) showed that 2 weeks of intensified HIT in the middle of a 6-week training period led to OR among trained cyclists, reporting reductions in MPO, maximal heart rate (HR_max_), $\dot{\text{V}}{\text{O}}_{2}\text{max}$ and cycling performance, as well as increases in global mood disturbance. In addition, Jürimäe et al. (2004) observed reductions in performance capacity, resting testosterone levels and recovery, as well as increases in stress levels, following 6 days of intensified training with trained rowers. Although block HIT studies have not typically monitored subjective markers of well-being, these findings highlight the importance of understanding the multi-dimensional responses to intense training interventions, especially given the potential for short-term OR to develop into the more chronic overtraining syndrome (Meeusen et al., 2013).

At present there is a lack of information regarding the physiological, psychological and performance-based responses of junior athletes performing block HIT, which would be relevant to coaches working in a variety of endurance sports. Therefore, the present investigation was designed to compare the effects of two polarized training interventions in well-trained junior male and female cross-country skiers. Nine HIT sessions were concentrated in the middle of a 3-week training period in the experimental intervention (BLOCK), while the same 9 HIT sessions were distributed evenly over the 3-week period in the control intervention (EVEN). Supplementary LIT and strength training were also matched across the two interventions, with only the organization of sessions differing. It was hypothesized that BLOCK would lead to a greater relative increase in $\dot{\text{V}}{\text{O}}_{2}\text{max}$ and 600-m time-trial (TT) performance compared with EVEN, despite covering less distance during the HIT sessions and attaining lower heart rates (HRs) during BLOCK compared with EVEN. Higher perceived exertion and stress, as well as reduced perceived recovery, were also expected during BLOCK compared with EVEN.

## Materials and methods

### Participants

Twenty well-trained cross-country skiers (10 males: age 17.6 ± 1.5 years, body mass 72.3 ± 4.8 kg, $\dot{\text{V}}{\text{O}}_{2}\text{max}$ 67.1 ± 2.6 mL·kg^−1^·min^−1^; 10 females: age 17.3 ± 1.5 years, body mass 61.1 ± 7.5 kg, $\dot{\text{V}}{\text{O}}_{2}\text{max}$ 54.2 ± 4.0 mL·kg^−1^·min^−1^) were recruited from two specialist Swedish ski schools. All participants had at least 6 years of experience racing in cross-country skiing and competed at a national level, while eight were also members of national junior development teams. Average endurance training volume was typically 500–750 h per year, or 9–14 h per week, and an additional 60–80 h of functional strength training was completed annually. Weekly training frequency was periodized, with 4–5 endurance sessions (6–8 h) completed per week during low volume periods and up to 12 endurance sessions (25 h) completed per week during high volume periods. After being informed of the aims and possible risks of the study the participants provided written informed consent to take part and informed parental consent was obtained for those aged under 18 years. The study was pre-approved by the Regional Ethical Review Board, Umeå University, Umeå, Sweden.

### Study overview

The study was conducted at the end of the cross-country ski racing season, from April to June. A crossover design was used, whereby one group of athletes (EVEN-BLOCK; 6 males and 7 females) completed 3 weeks of EVEN followed by 3 weeks of BLOCK, separated by a 4-day break, and the other group (BLOCK-EVEN; 4 males and 3 females) completed 3 weeks of BLOCK followed by 3 weeks of EVEN, also separated by a 4-day break (Table 1). As such, all 20 athletes performed both training interventions. The number of athletes in each group was not equal for the logistical reason that all HIT sessions needed to be completed on the same hill. Laboratory-based testing was carried out before and after the two interventions.

**Table 1:** An overview of the 53-day crossover study design incorporating two, 3-week training interventions (EVEN and BLOCK) flanked by pre- and post-testing.

	**Day**				
**Group**	**1–3**	**4–24**	**25–28**	**29–49**	**50–53**
EVEN-BLOCK (*n* = 13)	Test period 1	3-week EVEN	Test period 2	3-week BLOCK	Test period 3
BLOCK-EVEN (*n* = 7)		3-week BLOCK		3-week EVEN	

### Pre- and post-testing

**Figure 1:**
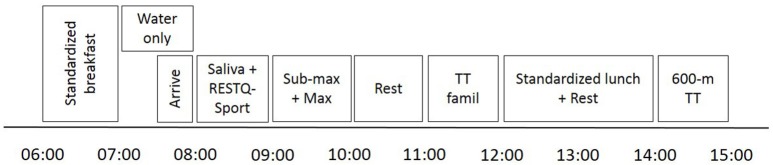
A schematic of the laboratory tests performed before and after the training interventions. RESTQ-Sport: 76-question recovery-stress questionnaire for athletes; Sub-max + Max: sub-maximal and maximal incremental tests; TT famil: 400-m familiarization to the 600-m time-trial (TT).

All participants attended the laboratory once during each test period to provide a resting saliva sample and to complete the 76-question recovery-stress questionnaire for athletes (RESTQ-Sport), as well as sub-maximal and maximal incremental tests and a 600-m TT (Figure 1). Participants were familiar with the sub-maximal and maximal treadmill protocols, having completed them routinely as part of their seasonal testing. The specific 600-m TT protocol was new to all participants, so a familiarization was included in the test battery. Participants arrived at the laboratory in a rested state having consumed a standardized breakfast. The sub-maximal and maximal incremental tests, as well as a familiarization to the 600-m TT, were completed in the morning prior to a standardized lunch, while the 600-m TT was completed after lunch. In addition to the main test day, muscle biopsies were taken on a separate day during each test period from a sub-group of 11 athletes aged 18 years and over (6 males: age 18.7 ± 0.8 years; 5 females: age 18.6 ± 0.9 years).

#### Saliva samples

Resting saliva samples were collected by passive drool (Beaven et al., 2008) on arrival at the laboratory, between 08:00–08:30 during each test period. The participants consumed only water in the 1 h prior to collection (Sperlich et al., 2012). Samples were collected in sterile tubes and stored at −20°C until analysis. After thawing and centrifuging at 2,000 rpm for 10 min, the samples were analyzed in duplicate and average values were used to determine cortisol, testosterone and immunoglobulin A (IgA) concentrations using commercial ELISA kits (Salimetrics LLC, Pennsylvania, USA), as described by Beaven et al. (2008).

#### RESTQ-Sport

The RESTQ-Sport was completed on arrival at the laboratory after providing a saliva sample. The questionnaire consists of 12 general scales and 7 additional sport-specific scales, with 4 questions per scale, and assesses the balance between perceived recovery and stress (Kellmann and Kallus, 2001). The total stress score corresponds to the sum of the scores of all of the stress subscales (7 general plus 3 sport-specific), while the total recovery score represents the sum of the scores of all of the recovery subscales (5 general plus 4 sport-specific). A general indicator of recovery-stress was calculated as the total recovery minus the total stress score (Kellmann and Kallus, 2001).

#### Sub-maximal and maximal incremental tests

Following the measurement of height and body mass (Seca 764, Hamburg, Germany), sub-maximal and maximal incremental tests were carried out on a motor-driven treadmill (Rodby RL 3000, Rodby, Vänge, Sweden) according to procedures previously described by McGawley and Holmberg (2014). Briefly, the diagonal roller-skiing technique and the same pair of pre-warmed roller-skis (Pro-Ski Classic C2, Sterners, Dala-Järna, Sweden) were used for both tests. The sub-maximal test was fixed at a 7° gradient and included a 4-min warm-up followed by four, 4-min continuous stages. Speeds differed for individuals depending on age, sex, and skiing ability, with the warm-up and first stage completed at 5.2–7.0 km/h and increases of either 0.8 or 1.0 km/h per stage to final speeds of 7.6–10.0 km/h. At the end of the sub-maximal test there was a 1-min break before participants commenced the maximal test. Depending on age, sex, and ability, the starting speed for the maximal test was 10, 11, or 12 km/h and the initial gradient was 3° or 4°. The gradient was then increased by 1° every minute, up to a maximum of 9°, after which speed was increased by 0.4 km/h every minute. The test was terminated when participants were unable to continue. Respiratory variables were measured using a mixed expired air procedure with an ergospirometry system (AMIS 2001 model C, Innovision A/S, Odense, Denmark) equipped with a flow meter. The gas analysers were calibrated with a high-precision mixture of 16.0% O_2_ and 4.0% CO_2_ (Air Liquide, Kungsängen, Sweden) and the flow meter was calibrated at three rates with a 3-L air syringe (Hans Rudolph, Kansas City, USA). The $\dot{\text{V}}{\text{O}}_{2}$ values were calculated from 10-s epochs and skiing economy is expressed as the 1-min steady-state value measured during the final minute of the second sub-maximal stage, while $\dot{\text{V}}{\text{O}}_{2}\text{max}$ is expressed as the highest 30-s average recorded over any three consecutive 10-s samples (i.e., a sliding average).

#### 600-m time-trial

A familiarization to the 600-m TT, which was limited to 400 m in order to minimize any impact of fatigue, was carried out on the same morning as the sub-maximal and maximal incremental tests, after at least 1 h of rest. The 600-m TT was then completed in the afternoon, following a standardized lunch and at least 2 h of rest, again according to the methods described by McGawley and Holmberg (2014). Following a 15-min warm-up the test protocol began with 100 m at a fixed speed (8.8 km·h^−1^ for the females and 10.8 km·h^−1^ for the males) to avoid over-pacing, followed by a self-paced maximal effort for the remaining 500 m. The treadmill gradient was fixed at 7° and the diagonal-stride technique was used throughout. The same motor-driven treadmill as that described in Section Sub-maximal and Maximal Incremental Tests was used, fitted with lasers that automatically increased or decreased the speed if the athlete moved to the front or rear of the belt, respectively, maintaining a constant speed otherwise (Swarén et al., 2013). Expired air was collected throughout using the procedures described above.

#### Muscle biopsies

Resting muscle biopsies were taken from the vastus lateralis 1–3 days before the main test day during each of the three test periods (see Table 1). After 10 min of supine rest the skin above the middle portion of the vastus lateralis was anesthetized with 2% lidocaine (B. Braun Medical, Danderyd, Sweden) and biopsies were taken using the needle technique with suction enhancement (Bergström, 1962; Hennessey et al., 1997). The tissue obtained was rapidly cleaned from blood and fat and divided into three parts. One part was mounted in an embedding medium (Tissue Tek® O.C.T. Compound) for subsequent histochemical analyses and frozen immediately in isopentane that was cooled to its freezing point in liquid nitrogen. The other two parts were immediately frozen in liquid nitrogen for subsequent enzyme and protein-content analyses. The samples were stored at −80°C until analyzed. For histochemical analysis, serial 10-μm cross-sections were cut in a cryostat at −20°C. Following preincubation at pH 4.3, 4.6, and 10.3, the sections were stained for myofibrillar ATPase at pH 9.4 and the muscle fibers were classified as type I, IIA, IIB, or IIC (Brooke and Kaiser, 1969). To visualize capillaries, the cross-sections were stained by the amylase-PAS procedure (Andersen, 1975). Computer image analysis (Leica QWin Runner V 3.5.1, Leica Microsystems, Bromma, Sweden) was performed to evaluate capillary density, fiber composition and fiber areas, as described by Kazior et al. (2016). Maximal enzyme activities of citrate synthase (CS), 3-hydroxyacyl CoA dehydrogenase (HAD) and phosphofructokinase (PFK) were carried out according to the procedures described by Opie and Newsholme (1967), Essén et al. (1975), Alp et al. (1976), respectively. The protein content of VEGF and PGC-1α were measured using western blots. Briefly, 20 mg of tissue was homogenized in 250 μl of RIPA buffer (Sigma) using glass/teflon homogenization. Following centrifugation at 13,000 g, total protein concentration of the supernatant was estimated using the PierceTM BCA Protein Assay kit (Thermo Fisher Scientific). Protein (25 μg) was separated by SDS-PAGE (NuPAGE® Bis-Tris Precast Gels, 4–12%), transferred to nitrocellulose membranes and detection was made using SuperSignalWestPico Chemiluminescent Substrate (Thermo Fisher Scientific). Antibodies used were Anti-PGC-1α Mouse mAb (4C1.3) (ST1202) and Anti-VEGF (Ab-3) Mouse mAb (14–124) (GF25) (Merck Millipore). Results are presented as ratios of VEGF or PGC-1α expression to a loading control, beta Actin (ab8227) (Abcam), ensuring equal loading on the gel (Ruas et al., 2012; Andrzejewski et al., 2015). Also, gel-to-gel variation was adjusted for using an internal standard.

### Training

#### EVEN and BLOCK interventions

The two, 3-week training interventions were developed in close cooperation with the coaches of the participating athletes. The EVEN intervention replicated a typical 3-week polarized training cycle, while BLOCK involved 1 week of LIT only, both before and after an intensified week of HIT only. The EVEN and BLOCK interventions were workload matched and included 7 and 9 LIT sessions, respectively (matched for total training time), as well as 9 HIT sessions and 6 functional strength sessions (Table 2).

**Table 2:** The number and distribution of low-intensity training (LIT), high-intensity interval training (HIT) and functional strength (STR) training sessions during three weeks of evenly-distributed (EVEN) and block (BLOCK) training.

	**EVEN**			**BLOCK**		
	**Week 1**	**Week 2**	**Week 3**	**Week 1**	**Week 2**	**Week 3**
LIT	2	3	2	4	0	5
HIT	3	3	3	0	9	0
STR	2	2	2	3	0	3

Durations of the LIT sessions ranged from 58 ± 4 to 127 ± 11 min (~60-, 90-, 105-, or 120-min sessions) and were completed at ~60–80% of HR_max_ as either skiing (~60% of all sessions, either roller- or on-snow skiing, depending on weather conditions), running (~35% of all sessions) or cycling (~5% of all sessions). The distribution of different LIT activities was similar between the two interventions. The HIT sessions were standardized at 75 min and consisted of a warm-up, 5 × 4-min intervals separated by 6 min of active recovery and a warm-down. The intervals were completed using the diagonal-stride cross-country skiing technique on the same pair of roller-skis throughout the study for each individual. The aim was to cover as much total distance as possible, as evenly as possible, over the five intervals within each session. Distance covered was measured to the nearest meter for all athletes during each interval for every HIT session. All intervals were completed on the same uphill asphalt slope (~12%/7°) and the active recovery involved a downhill jog back to the start. In the case of a problem with the roller-skis participants completed the session by running on the same uphill slope with poles (distance covered was not analyzed for running intervals). All HIT sessions were performed in groups of 6–8 athletes supervised by at least two researchers and one coach. A standardized 20-min warm-up, including a 2-min uphill interval on the training hill, was performed before each HIT session and a 15-min cool-down was performed afterwards. The 9 HIT sessions in week 2 of BLOCK were organized such that 2 sessions were completed on days 1, 2, and 5 (separated by at least 5 h of rest and a meal), 1 session was completed on days 3, 6, and 7, leaving day 4 as a rest day. The strength sessions completed throughout the study involved functional and complex exercises and were supervised as part of the athletes' regular training program.

#### Training loads

Heart rate was monitored for all LIT and HIT training sessions (Polar RS800CX, Polar Electro Oy, Kempele, Finland) and data were subsequently analyzed for the determination of average HR (HR_av_), HR_max_, session duration and time spent in each of the HR zones. The summated HR zone (sHRZ) method was used to quantify the HR-based training load for each session using the following five HR zones: 1 = 50–60% of HR_max_, 2 = 60–70% of HR_max_, 3 = 70–80% of HR_max_, 4 = 80–90% of HR_max_ and 5 = 90–100% of HR_max_ (Edwards, 1993; Foster et al., 2001). Cumulated time spent in each zone (in min) was multiplied by the zone value (i.e., 1–5) to obtain an overall sHRZ training load. The second approach to quantifying training load used a modification of the 0–10 category ratio rating scale (CR-10) originally presented by Borg (1982). As described by Foster et al. (2001), within 30 min after every training session participants responded to the simple question “How was your workout?” using a 10-point scale, with 0 and 10 corresponding to “rest” and “maximal,” respectively. The text on the scale was presented to the athletes in both English and Swedish. The session rating of perceived exertion (sRPE) training load was calculated by multiplying the 0–10 rating by the total session duration (in min) and expressed in arbitrary units (Foster et al., 2001). Total sRPE training load scores were calculated for each individual by summing the sRPE training loads for all LIT and HIT sessions during EVEN and BLOCK.

#### Recovery and stress measures

Following the warm-up prior to all LIT and HIT training sessions the participants reported their perceived recovery (pREC) on a scale from 0 to 10, with 0 and 10 corresponding to “very poorly recovered/extremely tired” and “very well recovered/highly energetic,” respectively (Laurent et al., 2011). The RESTQ-Sport was completed weekly on a rest day prior to starting each training week at a standardized time of day.

### Data analysis

The Statistical Package for the Social Sciences (SPSS, Version 22) was used to carry out statistical analyses. Interval and ratio data are expressed as mean ± standard deviation, while ordinal data (sRPE, pREC, and RESTQ-Sport) are expressed as median [range]. Paired *t*-tests were used to compare responses to HIT versus LIT and EVEN vs. BLOCK, while unpaired *t*-tests were used to compare responses between males and females. Two-way ANOVAs with repeated measures were used to identify the intervention (EVEN vs. BLOCK), time (pre- to post-training) and interaction effects. The magnitude of the training effect for EVEN vs. BLOCK was also assessed using effect size (ES), where differences of <0.2, <0.6, <1.2, and <2.0 are interpreted as trivial, small, moderate and large, respectively (Hopkins et al., 2009). For the interval and ratio data, two-way ANOVAs with *post-hoc* Tukey tests were used to identify interaction effects and differences between the interventions (EVEN vs. BLOCK) and training weeks and HR zones. For the ordinal data, Friedman tests were used to compare weeks within each intervention and Wilcoxon signed rank tests were used to compare pairwise responses to HIT versus LIT and EVEN versus BLOCK, while Mann-Whitney tests were used to compare responses between males and females. The level of statistical significance was set at *P* < 0.05.

## Results

### Changes from pre- to post-training

Pre- to post-training effects of EVEN and BLOCK are displayed in Table 3. There were no interaction effects between intervention and time for any of the performance, saliva or muscle biopsy variables, as demonstrated by the change (Δ) data (*P* > 0.05). Neither were there any significant intervention (group) effects (*P* > 0.05). However, there were significant time (i.e., pre- to post-training) effects, irrespective of group, for 600-m TT performance, resting testosterone concentration and testosterone:cortisol ratio, mean muscle fiber area, HAD and PFK activity and PGC-1α protein content.

**Table 3:** Mean ± SD pre- to post-training and change (Δ) data following three weeks of evenly-distributed (EVEN) or block (BLOCK) training.

	**EVEN**			**BLOCK**			**Δ**	
	**Pre**	**Post**	**Δ**	**Pre**	**Post**	**Δ**	***P*-value**	**ES**
***n** = **20***								
Skiing economy (O_2_ L·min^−1^)	2.83 ± 0.57	2.78 ± 0.57^*^	−0.06 ± 0.10	2.77 ± 0.58	2.79 ± 0.58	0.02 ± 0.16	0.117	0.79
VO_2_max(mL·kg^−1^ · min^−1^)	60.3 ± 7.2	61.2 ± 7.9	0.9 ± 2.6	61.4 ± 8.1	60.6 ± 8.2	−0.8 ± 2.4	0.071	0.67
600-m time-trial (s)^T^	187 ± 23	183 ± 25^*^	−3 ± 5	185 ± 23	184 ± 22	−1 ± 6	0.280	0.44
Resting cortisol (μg/dL)	0.45 ± 0.21	0.54 ± 0.30	0.09 ± 0.21	0.53 ± 0.32	0.48 ± 0.19	−0.05 ± 0.30	0.208	0.59
Resting testosterone (pg/mL)^T^	108 ± 75	96 ± 45	−12 ± 51	108 ± 53	86 ± 39	−20 ± 41	0.592	0.15
Resting testosterone:cortisol^T^	264 ± 203	216 ± 157	−48 ± 94	257 ± 190	218 ± 170	−39 ± 139	0.817	0.18
Resting IgA (μg/mL)	36 ± 34	76 ± 83	40 ± 96	72 ± 83	45 ± 43	−26 ± 69	0.081	0.68
***n** = **11***								
Capillary density (per mm^2^)	377 ± 31	379 ± 44	3 ± 34	385 ± 42	365 ± 36	−20 ± 39	0.253	0.66
Mean fiber area (μm^2^)^T^	4594 ± 761	4661 ± 764	68 ± 522	4596 ± 776	4968 ± 1000	372 ± 655	0.379	0.11
Type I (%)	66.2 ± 7.5	69.1 ± 6.7	2.9 ± 6.5	67.5 ± 6.5	67.2 ± 8.3	−0.3 ± 7.4	0.203	0.50
Type IIA (%)	25.2 ± 6.4	22.7 ± 5.6	−2.5 ± 4.8	24.1 ± 5.0	21.9 ± 7.9	−2.2 ± 8.3	0.904	0.07
Type IIB (%)	7.2 ± 4.1	7.2 ± 3.8	0.0 ± 3.2	7.5 ± 4.4	9.4 ± 5.9	1.9 ± 3.9	0.177	0.60
Type IIC (%)	1.4 ± 1.9	1.0 ± 2.4	−0.4 ± 3.4	1.0 ± 2.4	1.5 ± 2.6	0.5 ± 3.9	0.680	0.27
CS activity (μmol/min/g)	23.2 ± 2.9	22.5 ± 2.9	−0.7 ± 2.5	23.6 ± 2.7	22.5 ± 1.7	−1.1 ± 2.1	0.743	0.15
HAD activity (μmol/min/g)^T^	7.4 ± 1.0	7.4 ± 1.1	0.0 ± 1.1	7.6 ± 1.1	6.9 ± 0.9^*^	−0.7 ± 1.0	0.317	0.60
PFK activity (μmol/min/g)^T^	21.7 ± 2.9	20.7 ± 2.1	−1.0 ± 2.3	20.8 ± 2.1	20.1 ± 2.4	−0.7 ± 1.9	0.753	0.14
VEGF protein content (AU)	20.0 ± 9.3	15.4 ± 10.7	−4.7 ± 15.2	16.5 ± 10.9	19.9 ± 15.5	3.5 ± 12.4	0.235	0.53
PGC-1α protein content (AU)^T^	0.07 ± 0.02	0.09 ± 0.02^*^	0.02 ± 0.02	0.08 ± 0.01	0.11 ± 0.04^*^	0.03 ± 0.04	0.862	0.11

CS, Citrate synthase; HAD, 3-hydroxyacyl CoA dehydrogenase; PFK, phosphofructokinase; AU, arbitrary units.

T Significant time (pre- to post-training) effect, irrespective of intervention (P < 0.05);

* *Significantly different from pre-training (P < 0.05)*.

### Adherence to training

In total the athletes completed 97 ± 6% (range: 90–100%) of the HIT sessions during EVEN and all (i.e., 100 ± 0%) of the HIT sessions during BLOCK and 91 and 99% of these sessions, respectively, were performed using diagonal roller-skiing. A total of 95 ± 4% (range: 90–100%) and 98 ± 4% (range: 90–100%) of the LIT sessions were completed during EVEN and BLOCK, respectively.

### Responses to training: LIT vs. HIT

A description of the HR and sRPE responses during LIT and HIT for EVEN and BLOCK combined (i.e., independent of intervention type) is presented in Table 4 for all participants, and for males and females separately.

**Table 4:** Mean ± SD average heart rate (HR_av_), maximal heart rate (HR_max_), % of the total training time spent in zones 1–5 (Z1–Z5) and summated heart rate zone (sHRZ) scores and median [range] session rating of perceived exertion (sRPE) scores during low-intensity training (LIT) and high-intensity interval training (HIT).

			**All participants**	**Males**	**Females**
HR_av_ (beats·min^−1^)		LIT	133 ± 9	130 ± 7	136 ± 10
		HIT	153 ± 8^**^	152 ± 7^**^	155 ± 9^**^
HR_max_ (beats·min^−1^)		LIT	159 ± 8	156 ± 3	161 ± 10
		HIT	191 ± 6^**^	191 ± 6^**^	192 ± 7^**^
% of total training time spent in each zone	Z1	LIT	21 ± 15	25 ± 15	16 ±15
		HIT	11 ± 5^**^	11 ± 6^*^	10 ± 5
	Z2	LIT	48 ± 13	56 ± 13	40 ± 7^††^
		HIT	26 ± 8^**^	30 ± 6^**^	21 ± 8^†^
	Z3	LIT	29 ± 18	18 ± 10	40 ± 18^††^
		HIT	25 ± 7	22 ± 8	27 ± 6^*^
	Z4	LIT	2 ± 3	1 ± 1	4 ± 3^††^
		HIT	19 ± 4^**^	19 ± 4^**^	19 ± 5^**^
	Z5	LIT	0 ± 0	0 ± 0	0 ± 0
		HIT	20 ± 8^**^	17 ± 6^**^	23 ± 8^**^
Total sHRZ score		LIT	3,196 ± 509	2,896 ± 280	3,496 ± 517^††^
		HIT	4,227 ± 377^**^	4,069 ± 303^**^	4,385 ± 390^**^
Total sRPE score		LIT	5,440 [3,735–6,983]	4,230 [3,735–6,489]	6,186 [3,989–6,983]^†^
		HIT	10,463 [9,225–11,592]^**^	10,200 [9,225–11,550]^*^	10,725 [9,488–11,592]^*^

Significantly different from LIT:

* *P < 0.05*,

** *P < 0.001*.

Significantly different from the males:

† *P < 0.05*,

†† *P < 0.01*.

### Responses to training: even vs. block

#### Training time for LIT

The LIT durations for the three separate weeks differed during EVEN and BLOCK (Table 5), while total time spent performing LIT did not differ between the two interventions (742 ± 33 and 754 ± 28 min for EVEN and BLOCK, respectively; *P* = 0.218).

**Table 5:** Mean ± SD weekly durations (min) for low-intensity training (LIT) during three weeks of evenly-distributed (EVEN) and block (BLOCK) training.

	**Week 1**	**Week 2**	**Week 3**
EVEN	231 ± 29	272 ± 5	239 ± 11
BLOCK	319 ± 9^*^	0 ± 0^*^	435 ± 26^*^

Significantly different from EVEN:

* *P < 0.001*.

#### Performance during HIT

Average distances covered during each of the 5 × 4-min intervals were similar during EVEN and BLOCK and are presented in Table 6 for all participants, as well as the males and females.

**Table 6:** Mean ± SD average distance covered (m) during the 4-min high-intensity intervals during three weeks of evenly-distributed (EVEN) and block (BLOCK) training.

	**All participants**	**Males**	**Females**
EVEN	740 ± 71	795 ± 38	675 ± 35^*^
BLOCK	736 ± 75	792 ± 46	671 ± 43^*^

Significantly different from the males:

* *P < 0.001*.

#### Heart rate zones

**Figure 2:**
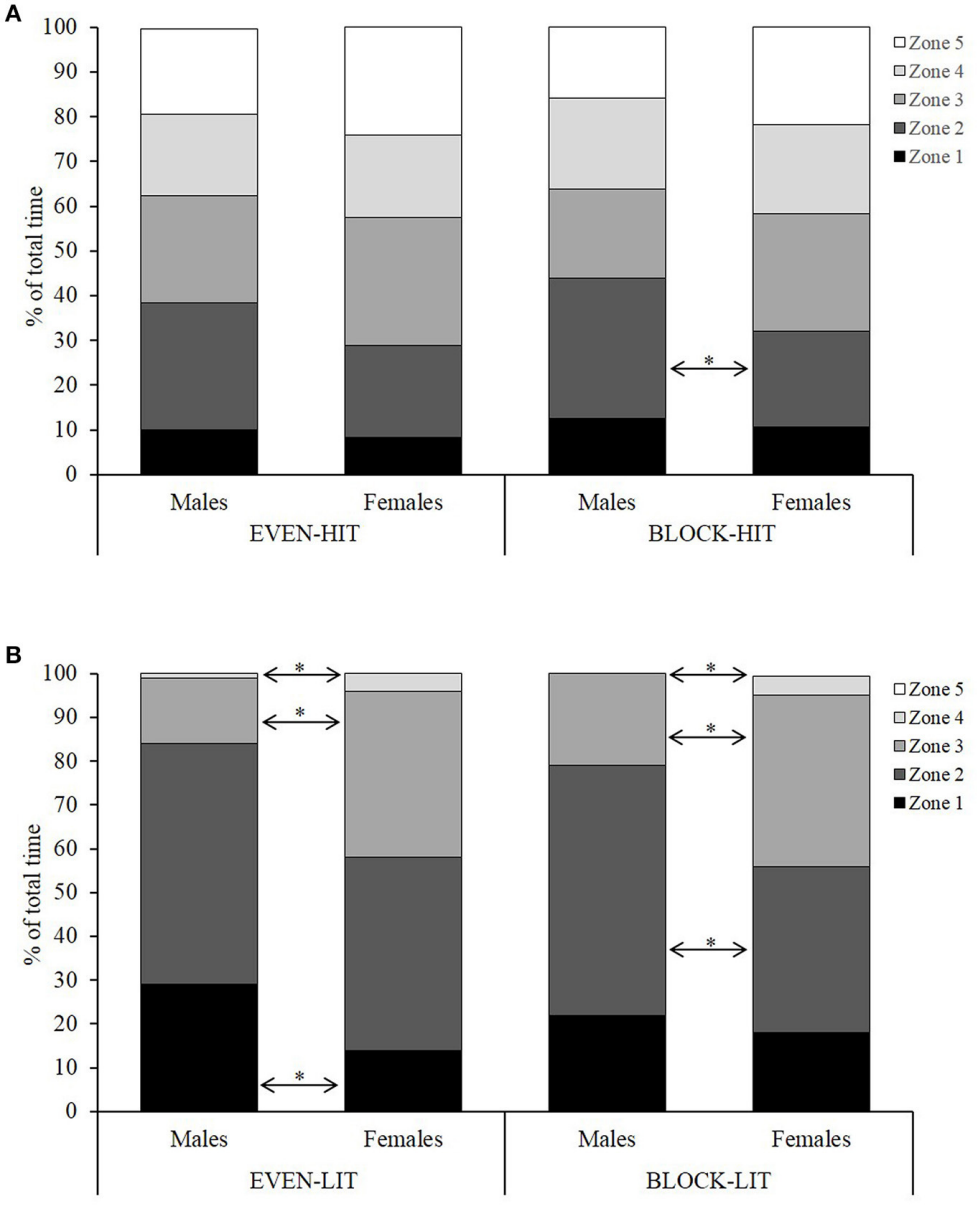
Percentage of the total time spent by the male and female skiers in the five different heart rate zones during the HIT **(A)** and LIT **(B)** sessions for the evenly-distributed (EVEN) and block (BLOCK) training interventions. Significantly different between the males and females: ^*^P < 0.05.

The % of total time spent in each of the five HR zones during HIT and LIT in EVEN and BLOCK for both the males and females is displayed in Figure 2. Overall there were no significant differences between interventions in the proportion of total training time spent in each of the HR zones (~16, 37, 27, 10, and 10% of total time in zones 1–5, respectively, for both interventions). Furthermore, the total sHRZ scores did not differ between EVEN and BLOCK (3,739 ± 440 and 3,684 ± 449, respectively; *P* = 0.329). However, the females demonstrated a significantly higher total sHRZ score during EVEN compared with the males (4,012 ± 392 and 3,466 ± 298, respectively; *P* = 0.003) and a non-significant tendency for the same difference during BLOCK (3,869 ± 520 and 3,498 ± 281, respectively; *P* = 0.067).

#### Perceived exertion and recovery

The median [range] sRPE and pREC scores during each of the separate training weeks for EVEN and BLOCK are displayed in Table 7. The total sRPE training loads did not significantly differ between EVEN and BLOCK (7,751 [5,758–9,462] and 8,127 [6,338–10,085], respectively; *P* = 0.286). However, the females demonstrated a significantly higher total sRPE training load for EVEN compared with the males (8,076 [6,140–9,462] and 7,530 [5,758–8,155], respectively; *P* = 0.031) and a non-significant tendency for the same difference during BLOCK (8,423 [7,337–8,948] and 7,740 [6,338–10,085], respectively; *P* = 0.190). The pREC prior to each of the HIT sessions was significantly improved for EVEN compared with BLOCK for all participants (6 [3–10] vs. 4 [1–8], respectively; *P* < 0.001), as well as for the males (6 [3–10] vs. 4 [1–8], respectively; *P* < 0.001) and the females (6 [3–8] vs. 4 [1–8], respectively; *P* < 0.001).

**Table 7:** Median [range] session rating of perceived exertion (sRPE) and perceived recovery (pREC) scores during the individual evenly-distributed (EVEN) and block (BLOCK) training weeks.

		**sRPE**			**pREC**		
		**All**	**Males**	**Females**	**All**	**Males**	**Females**
EVEN	Week 1	7 [1–10]^**^	7 [1–9]^**^	7 [2–10]^**^	6 [3–9]	6 [4–9]	6 [3–8]
	Week 2	6 [1–9]^**^	4 [1–9]^**^	7 [2–9]^**^	6 [3–10]^**^	7 [3–10]^**^	6 [3–8]^**^
	Week 3	6 [2–10]^**^	5 [2–10]^**^	6 [3–10]^**^	5 [3–8]^**^^†^	6 [3–8]^†^	5 [3–8]^**^^†^
	All weeks	6 [1–10]	5 [1–10]	6 [2–10]	6 [3–10]	6 [3–10]	6 [3–8]
BLOCK	Week 1	3 [2–6]^††^	3 [2–5]^††^	3 [2–6]^††^	6 [3–9]^††^	7 [5–9]^††^	6 [3–9]^††^
	Week 2	8 [2–10]	8 [2–10]	8 [4–10]	4 [1–8]	4 [1–8]	4 [1–8]
	Week 3	4 [2–7]^††^	3 [2–6]^††^	4 [2–7]^††^	7 [3–9]^††^	7 [3–9]^††^	7 [4–8]^††^
	All weeks	6 [2–10]	5 [2–10]	7 [2–10]	5 [1–9]	6 [1–9]	5 [1–9]

*sRPE, assessed using a modified CR-10 scale with 0 and 10 corresponding to rest and maximal, respectively; pREC, assessed on a scale with 0 and 10 corresponding to very poorly recovered/extremely tired and very well recovered/highly energetic, respectively*.

Significantly different from the corresponding BLOCK week:

** *P < 0.001*.

Significantly different from week 2:

† *P < 0.01*,

†† *P < 0.001*.

#### Recovery-stress state

The overall recovery-stress state (as measured by the RESTQ-Sport) did not significantly differ between EVEN and BLOCK (*P* = 0.510), but was significantly lower (indicating a less recovered/more stressed state) after week two for BLOCK compared with EVEN (14 [1–28] vs. 18 [5–35], respectively; P = 0.033). In addition, the females had a significantly lower recovery-stress state compared with the males during BLOCK after week two (11 [1–20] vs. 21 [13–28], *P* < 0.001) and three (15 [0–23] vs. 22 [11–30], *P* = 0.014).

## Discussion

The present investigation has shown that a 3-week polarized training intervention incorporating a block distribution of HIT is well-tolerated by a group of male and female junior cross-country skiers. In contrast to the hypothesis, distance covered during HIT was not lower during BLOCK compared with EVEN. In addition, despite weekly differences, overall total sHRZ scores, time spent in each of the HR zones, perceived exertion scores and training loads, perceived recovery and the overall recovery-stress state were not different following BLOCK compared with EVEN. In terms of pre- to post-training, no differences were observed between the changes in any of the performance or physiological measures following the two interventions (see Table 3). However, there were significant time effects for 600-m TT performance and a number of hormonal and muscle biopsy markers, irrespective of intervention. Based on these findings the current study does not support block-distributed HIT as a superior method to evenly-distributed HIT in terms of enhancing physiological or performance adaptions.

Block training is based on the theory that a period of highly concentrated, specialized loading will generate an increase in the training stimulus such that, following a period of recovery, work capacity and performance will increase due to a super-compensation (Issurin, 2008, 2010). At the same time, aerobic-based HIT has been shown to maximize the time spent exercising close to $\dot{\text{V}}{\text{O}}_{2}\text{max}$, which is considered the most effective stimulus for developing the oxygen transport and utilization systems (Billat, 2001; Midgley et al., 2006). A number of studies have combined the concepts of block training and HIT, showing positive improvements in $\dot{\text{V}}{\text{O}}_{2}\text{max}$, MPO, sub-maximal power output and TT performance among trained athletes following 13–15 HIT sessions completed within 10–14 days (Stølen et al., 2005; Breil et al., 2010; Wahl et al., 2013). While these studies highlight the potential benefits of blocking HIT, they did not compare different types of training organization. Therefore, the observed effects may simply be due to the training stimulus, *per se*, rather than the specific distribution of HIT sessions. More recently, Rønnestad and colleagues have completed a series of studies comparing block- with more evenly-distributed HIT interventions (Rønnestad et al., 2014a,b, 2016). Greater improvements in $\dot{\text{V}}{\text{O}}_{2}\text{max}$ were reported for trained cyclists following block- (5 HIT sessions in week 1, 1 HIT session in weeks 2–4) compared with evenly- (2 HIT sessions per week) distributed HIT (Rønnestad et al., 2014a,b). Among competitive cross-country skiers and biathletes, by contrast, improvements in $\dot{\text{V}}{\text{O}}_{2}\text{max}$ were not greater following block- versus evenly-distributed HIT, but the block training group improved MPO and sub-maximal power output to a greater extent than the even training group (Rønnestad et al., 2016). The current study aimed to investigate the potential mechanisms for the superior effects of block- compared with evenly-distributed HIT by systematically monitoring the daily responses to training (through HR, sRPE, recovery and performance measures), as well as by examining the peripheral adaptations in the muscle through pre- and post-intervention muscle biopsies.

Despite a more intense HIT stimulus applied in the current study compared with Rønnestad et al. (2014a,b); Rønnestad et al. (2016), no significant differences were observed between BLOCK and EVEN for any of the variables measured pre- to post-training (i.e., skiing economy, $\dot{\text{V}}\text{O}2\text{max}$, TT performance, resting salivary markers or muscle biopsy markers). This could be due to a number of reasons relating to the study design. Firstly, the current study was conducted at the end of the cross-country season, whereas all other published block training studies have been completed during pre-season (Breil et al., 2010; Wahl et al., 2013; Rønnestad et al., 2014a,b, 2016). Since Losnegard et al. (2013) have shown that cross-country skiers perform more HIT and less LIT toward the end of the competitive season, it is possible that the timing and resulting training status of the athletes in the present study affected the efficacy of the BLOCK intervention. Another factor could be the lack of any HIT sessions in the final training week during BLOCK. Anecdotally, athletes in the current study reported feelings of lethargy as a result of only having performed LIT in the week prior to laboratory testing. This was not the case following EVEN, whereby three HIT sessions had been performed in the week prior to testing. Bosquet et al. (2007) refer to maintenance of training intensity during an optimal taper and in support of this, Rønnestad et al. (2014a,b, 2016) prescribed at least 1 HIT session per week during recovery following their block intervention. Therefore, the maintenance of some HIT sessions in the weeks following the overload period may be critical in detecting beneficial effects of block training.

As well as investigating a range of pre- to post-training markers to assess the efficacy of BLOCK compared with EVEN, a large focus of the current study was directed toward examining the responses during training, in order to explain any potential differences between the two interventions. It was expected that less total distance would be covered during the HIT sessions in BLOCK compared with EVEN, due to the reduced recovery between sessions and subsequent accumulation of fatigue. For instance, power output produced by endurance-trained athletes has been observed to be lower during a second session of HIT performed on the same day compared to on a separate day (Yeo et al., 2008). Unexpectedly, however, the average distance covered during the 5 × 4-min intervals was similar between the two interventions in the present study (~740 m per interval). In contrast to Yeo et al. (2008), who allowed only 2 h of rest and water consumption between sessions, participants in the current study rested for 4–5 h and ate a meal between any two HIT sessions on the same day. Therefore, longer recovery and energy replacement may help to maintain performance when completing two HIT sessions on the same day. Alternatively, the whole-body nature of cross-country skiing exercise may lead to reduced local fatigue and allow training intensities to be maintained during a second training session within a day. The relatively long recovery duration of 6 min between each interval, which resulted from the logistical requirement for athletes to jog back down the hill after each interval, may also have enabled the maintenance of work done over the five repetitions. In fact, as little as 2 min of recovery between 4-min HIT bouts has been shown to be sufficient in maintaining performance in a set of repeated intervals, although a higher average oxygen consumption was possible during intervals with a 2- vs. 4-min recovery period (Seiler and Hetlelid, 2005).

Similar to distance covered, it was also expected that the athletes in the current study would attain lower HRs during BLOCK compared with EVEN, due to the more concentrated training load and reduced recovery between HIT sessions. This response has previously been demonstrated for competitive cyclists during maximal exercise following a period of intensified training predominantly consisting of HIT (Jeukendrup et al., 1992). However, no differences were identified in total sHRZ scores or the time spent in each of the HR zones during EVEN compared with BLOCK. With no differences observed for the group as a whole, further analyses were conducted to compare differences in the responses between the males and females. Interestingly, the females demonstrated a higher sHRZ score than the males during EVEN, with a non-significant tendency for the same difference during BLOCK. This appears attributable to the fact that the females spent more time in zones 3 and 4 during the LIT sessions, while the males spent more time in zones 1 or 2. While only speculative, it is possible that some of the females worked relatively harder during the LIT sessions in order to “keep up” with other members of the training group. While the athletes typically trained with others of a similar standard, a group session may have put pressure on the weaker members (often females) to work at a higher relative intensity than the stronger members (often males). Furthermore, any common undulating training routes, where specific techniques (and therefore velocities, to a certain extent) are employed on given inclines, would also likely lead to higher relative intensities among the females due to lower maximal aerobic capacities. In addition to differences during the LIT sessions, the males also spent significantly more time in zone 2 during the BLOCK HIT sessions, with the females tending to spend more time in the higher HR zones. This indicates a more rapid HR recovery among the males between intervals. Overall these findings highlight the need for coaches to carefully monitor the internal loads (i.e., HR responses) of individuals within a training group, especially in mixed-sex groups, to ensure that specified training targets are achieved.

Subjective measures have been reported to be more sensitive and consistent than objective measures when monitoring changes in athlete well-being in response to training (Saw et al., 2016), hence the use of sRPE, pREC, and RESTQ-Sport in the current study. An analysis of the separate weeks highlighted clear distinctions in the differing demands during BLOCK and EVEN, with sRPE scores significantly higher and pREC scores significantly lower during week two of BLOCK compared with weeks one and three, as well as compared with week two of EVEN. In addition, pREC was improved prior to the HIT sessions during EVEN compared to BLOCK. This indicates an improved readiness to train when HIT sessions are spread out over 3 weeks rather than being condensed into 1 week. The extreme training load prescribed in week two of BLOCK was the basis for hypothesizing that perceived exertion would have been higher and perceived recovery would have been lower after BLOCK compared with EVEN. However, results showed no differences in sRPE scores, sRPE training loads or average pREC scores after the two, 3-week interventions. Interestingly, and consistent with the sHRZ data, the females demonstrated a significantly higher sRPE training load during EVEN compared with the males and a tendency for the same difference during BLOCK. This may be for a similar reason to that previously proposed; that is, higher relative intensities and more time spent in higher HR zones may have resulted in a higher perception of effort among the females compared with the males.

Previous studies investigating periods of intensified training among endurance athletes have shown short-term reductions in recovery and well-being, as well as increases in mood disturbance and stress levels (Jeukendrup et al., 1992; Halson et al., 2002; Jürimäe et al., 2004; Coutts et al., 2007). Since an excess of stress can result in long-term performance decline that is manifested as overtraining, or non-functional OR (Meeusen et al., 2013), there was a potential risk for the young athletes in the current study performing so many HIT sessions within 1 week. Therefore, the RESTQ-Sport, which has been identified as a useful tool for monitoring perceived stress and recovery among athletes (Saw et al., 2016), was administered weekly (in contrast to the session-based pREC scale). Despite a significant difference during week two, the overall recovery-stress state was not different following BLOCK compared with EVEN. A rapid restoration of the recovery-stress state is consistent with previous findings that have shown global mood state to recover to baseline after 4–6 days of easy training (Halson et al., 2002). Therefore, it seems that non-functional OR may be avoided by limiting the duration of the intense training period and allowing sufficient recovery afterwards. An interesting and unexpected finding in the current study was that the females demonstrated lower recovery-stress states compared with the males, with the largest differences observed after week two of BLOCK. Thus, this study provides novel data to suggest that female athletes are more vulnerable than males to the stressors associated with block-distributed HIT within a polarized microcycle, perhaps due to higher internal workloads during training sessions.

The current study is the first to have comprehensively compared the responses during, and effects of, two polarized training models differing only in the distribution of training sessions. Due to the high adherence rates (90–100% of sessions completed by all individuals during HIT and LIT), the results may be considered a true representation of the prescribed interventions. Findings have shown distinct demands on the athletes during the three separate weeks of EVEN and BLOCK, demonstrated by the significant weekly differences in time spent performing LIT and HIT, perceived exertion and recovery scores and recovery-stress states. Despite this, the overall responses during the two interventions were typically similar in terms of performance and subjective measures (i.e., distance covered during HIT, session ratings of perceived exertion, perceived recovery and recovery-stress states). Moreover, changes pre- to post-training did not differ between EVEN and BLOCK. Some limitations of the present study may be related to the experimental design, specifically the lack of any HIT sessions following the intensified training week, the relatively short duration between the intensified training week and follow-up laboratory tests, the short time period (3 weeks) over which the interventions were prescribed and/or the relatively long recovery duration (6 min) between the 4-min HIT intervals. Nevertheless, a novel aspect of the study is the comparison between males and females, which revealed some real practical issues for coaches whereby the females typically demonstrated higher HR responses and sRPE scores, as well as higher stress scores and lower recovery-stress states, compared to the males. In light of these specific differences, future research may be directed toward investigating how higher internal training loads, perceived exertion and subjective recovery-stress states in females influence long-term training adaptations and potential OR or overtraining. In conclusion, the current study has shown that well-trained junior cross-country skiers are able to complete 9 HIT sessions within 1 week without compromising total work done or experiencing greater stress or reduced recovery in comparison to completing 3 HIT sessions per week over 3 weeks. However, a short training intervention using block-distributed HIT is not supported as being superior to evenly-distributed HIT when applied to well-trained, junior cross-country skiers.

## Ethics statement

This study was carried out in accordance with the recommendations of the Regional Ethical Review Board (Umeå Sweden) with written informed consent from all subjects (and informed parental consent for those aged under 18 years). All subjects gave written informed consent in accordance with the Declaration of Helsinki. The protocol was approved by the Regional Ethical Review Board, Umeå University, Umeå Sweden.

## Author contributions

KM, EJ, and HH made substantial contributions to the conception and design of the work while KM, EJ, ZK, KS, EB, OH, and HH all made substantial contributions to the acquisition, analysis and interpretation of data for the work. All authors (KM, EJ, ZK, KS, EB, OH, and HH) were involved in the drafting and critical revision of the work, as well as the final approval of the version to be published, and agree to be accountable for all aspects of the work.

### Conflict of interest statement

The authors declare that the research was conducted in the absence of any commercial or financial relationships that could be construed as a potential conflict of interest.
